# Author Correction: Molecular quantitative trait loci in reproductive tissues impact male fertility in cattle

**DOI:** 10.1038/s41467-024-45727-9

**Published:** 2024-02-19

**Authors:** Xena Marie Mapel, Naveen Kumar Kadri, Alexander S. Leonard, Qiongyu He, Audald Lloret-Villas, Meenu Bhati, Maya Hiltpold, Hubert Pausch

**Affiliations:** 1 Animal Genomics, ETH Zurich, Universitatstrasse 2, 8092 Zurich, Switzerland; 2 Present Address: Roslin Institute, The University of Edinburgh, Easter Bush Campus, Midlothian, EH25 9RG UK; 3 Present Address: GenPhySE, Université de Toulouse, INRAE, ENVT, 31326 Castanet Tolosan, France

**Keywords:** Agricultural genetics, Data integration, Infertility, Quantitative trait, Gene expression analysis

Correction to: *Nature Communications* 10.1038/s41467-024-44935-7, published online 22 January 2024

In this article the grant number 310030_185229 relating to the Swiss National Science Foundation for Hubert Pausch was omitted. The original article has been corrected.

