# Supplementary material for: Molecular quantitative trait loci in reproductive tissues impact male fertility in cattle
Source: Nat Commun. 2024 Jan 22;15:674. doi: 10.1038/s41467-024-44935-7 (PMC10803364; doi:10.1038/s41467-024-44935-7)
Supplement: Supplementary file 1 — Supplementary Information [file 41467_2024_44935_MOESM1_ESM.pdf]

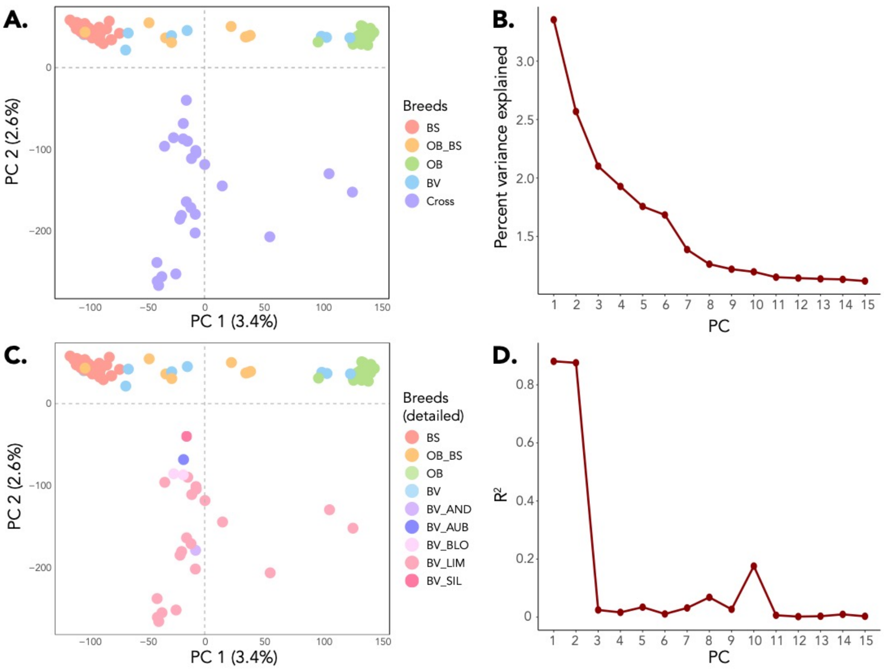

Supplementary Figure 1. Ancestry of 118 bulls from the molQTL cohort. A.) Principal components analysis (PCA) of LD-pruned WGS variants (366,090 variants total), with colors corresponding to the breed reported in the breed herdbook (BS = Brown Swiss; OB_BS = cross between Original Braunvieh and Brown Swiss; OB = Original Braunvieh; BV = Braunvieh) or "Cross". B.) The percent variance explained for the first 15 principal components (PCs) of the genotype PCA. C.) PCA of WGS variants as in A), but with colors corresponding to the specific breed reported. Abbreviations of the cross breeds are the following: AND = other (unknown); AUB = Aubrac; BLO = Blonde d'Aquitaine; LIM = Limousin; SIL = Silian (Simmental, Limousin and Angus). D.) Correlation between the PCs of the genotype PCA and the breed reported.

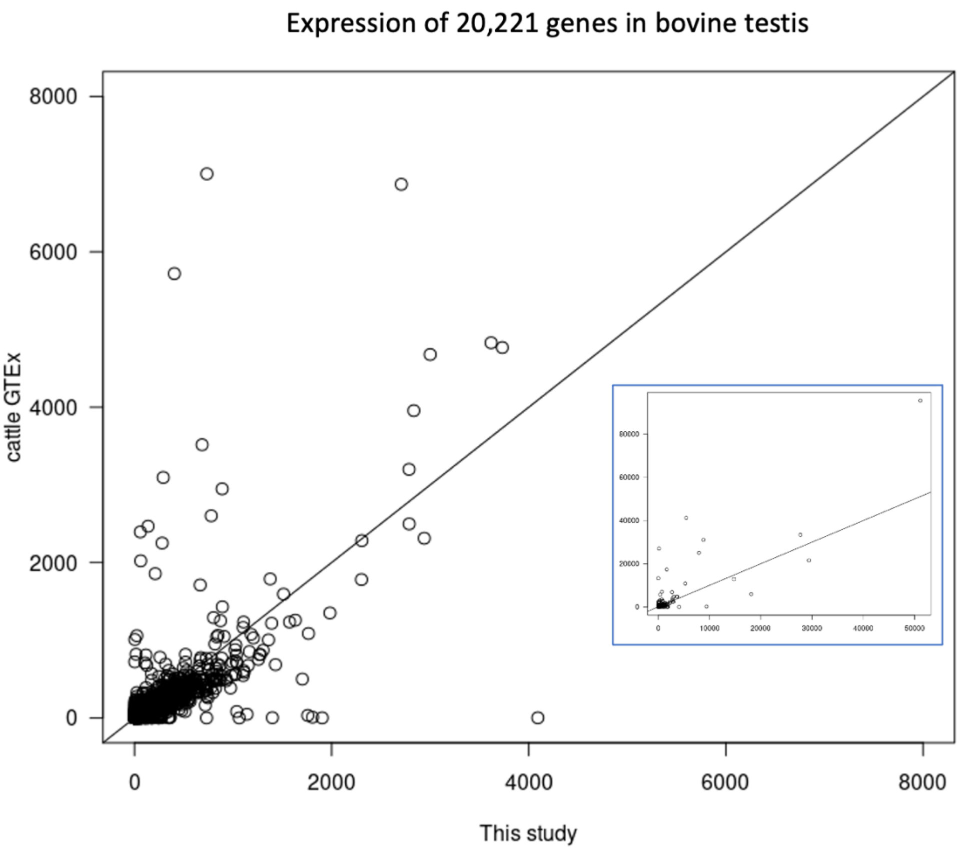

Supplementary Figure 2. Comparison of testis gene expression between this study and cattle GTEx. Scatterplot of TPM values for 20,221 genes that are part of the current study and cattle GTEx [1]. The axes are truncated at 8000. The solid line is a line through the origin. The inset shows the same plot without truncated axes. TPM estimates from cattle GTEx were obtained from the version 1.2 dataset deposited at zenodo (https://zenodo.org/record/7560235). Samples were filtered based on meta data to keep only post-pubertal taurine bulls. The following samples were retained from cattle GTEx: SRS3309609, SRS3309607, SRS3309608, SRS3021383, CRS013209, CRS013202, CRS013216, CRS013223, SRS734278, SRS485384.

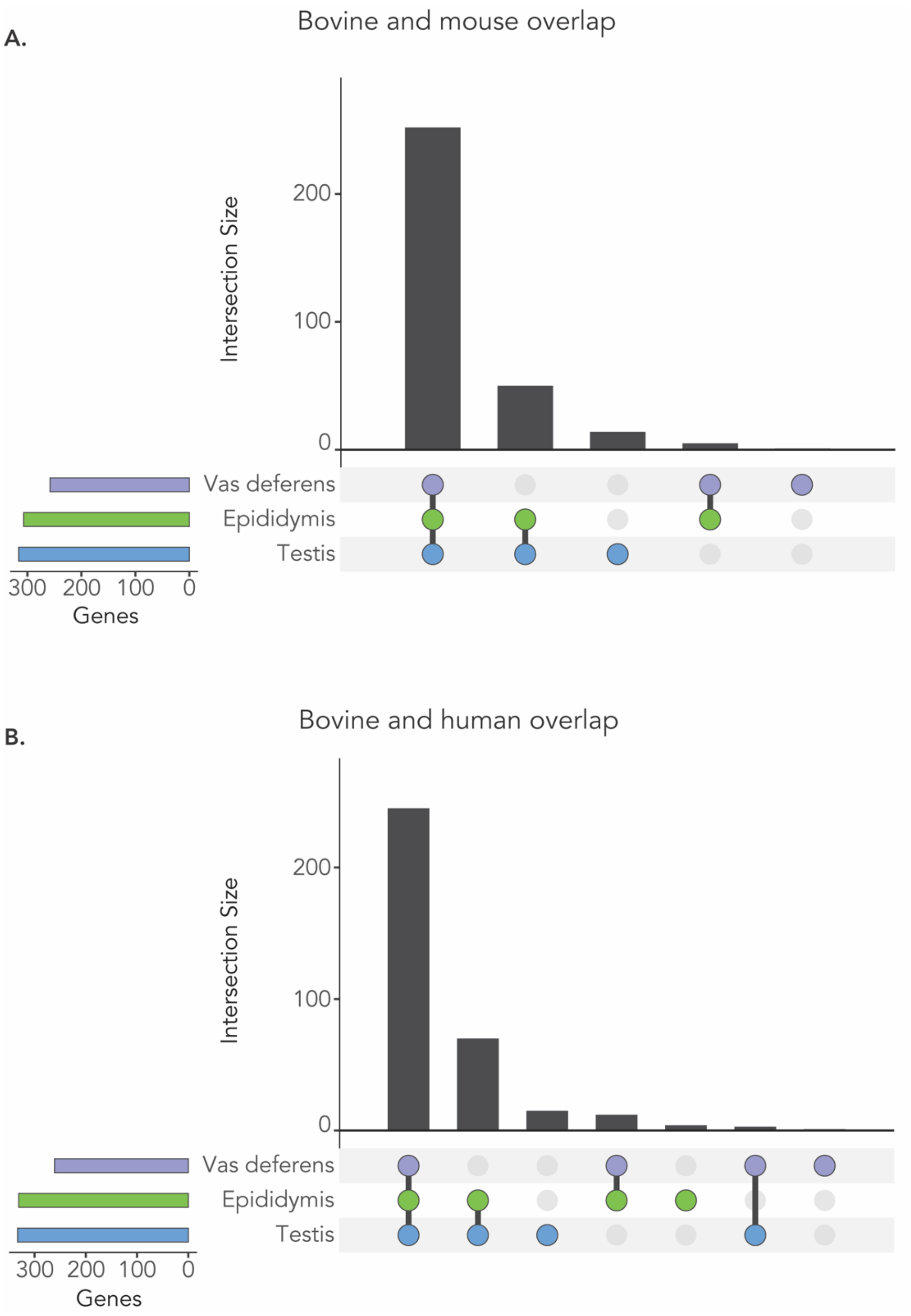

Supplementary Figure 3. Overlap of genes expressed in three bovine reproductive tissues with male reproductive tract-specific expressed genes in humans and mice. We were able to unambiguously identify bovine orthologs for 380 of 720 reproductive tract-specific expressed genes in humans, and for 329 of 699 reproductive tract-specific expressed genes in mice [2]. A) UpsetR plot showing the expression pattern of the bovine orthologs of reproductive tract-specific expressed genes in mice. B) UpsetR plot showing the expression pattern of the bovine orthologs of reproductive tract-specific expressed genes in human.

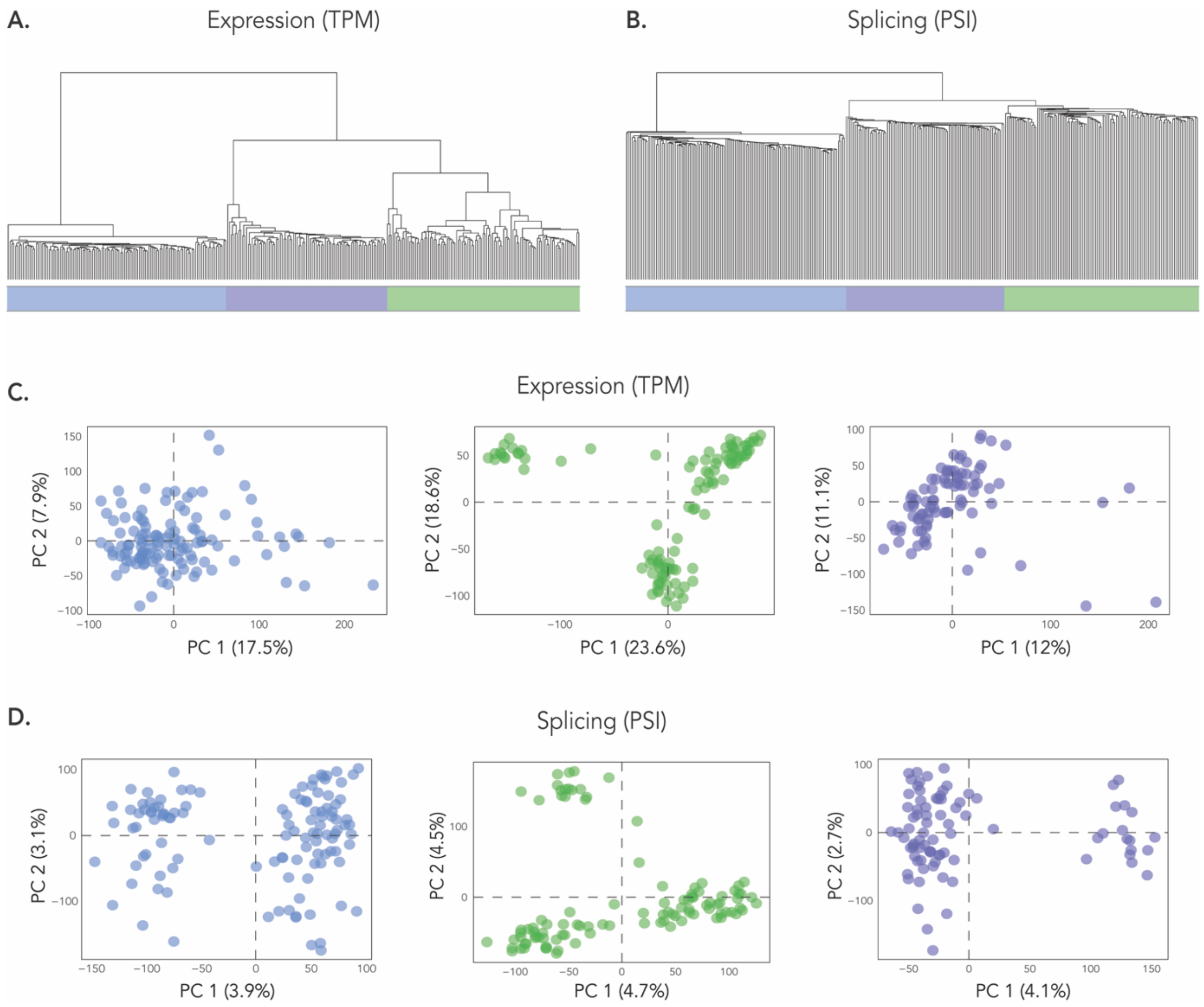

Supplementary Figure 4. Expression and splicing across male reproductive tissues. A.) Hierarchical clustering from the r package *dendextend* for the normalized expression values (TPM) across the three tissues. Colored bars correspond to a sample's tissue type (testis: blue; epididymis: green; vas deferens: purple). B.) Hierarchical clustering for the normalized splicing values (PSI) across the three tissues. Colored bars correspond to a sample's tissue type. C.) Principal components analysis (PCA) of TPM values within individual tissues. The left plot is testis samples (blue), the middle plot is epididymis samples (green), and the right plot is vas deferens samples (purple). D.) PCA of PSI values within individual tissues. The left plot is testis samples (blue), the middle plot is epididymis samples (green), and the right plot is vas deferens samples (purple).

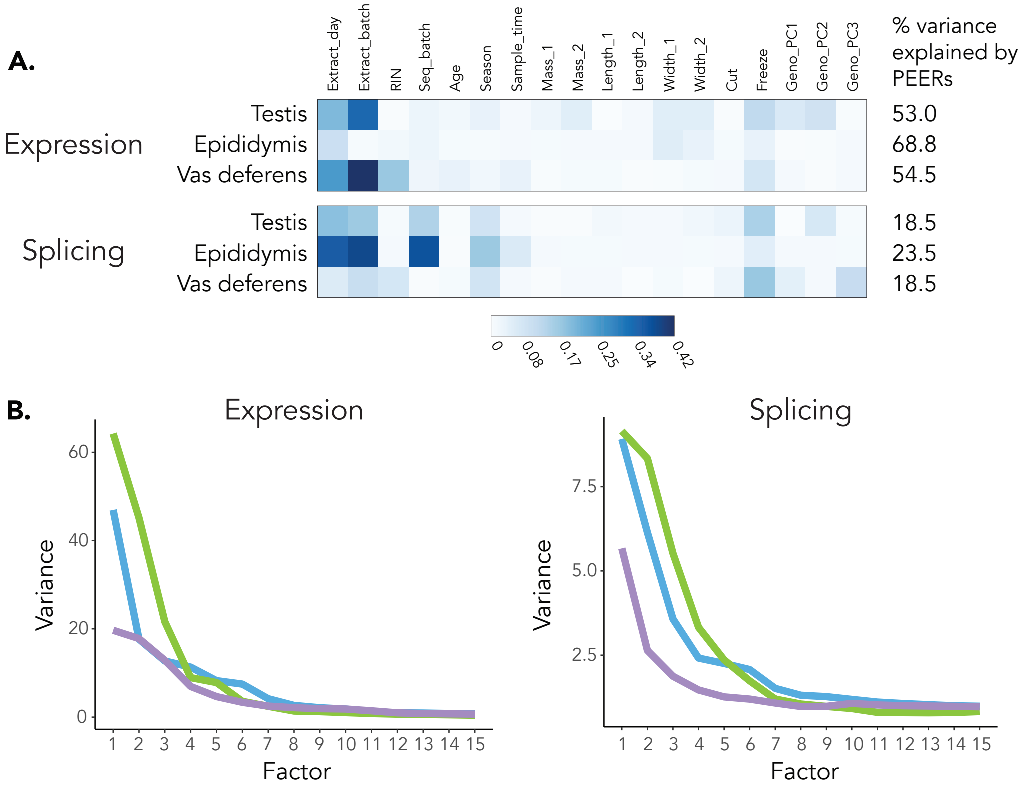

Supplementary Figure 5. PEER factors for expression and splicing phenotypes across tissues. A.) Correlation between PEER factors and covariates for expression and splicing phenotypes across all tissues. The covariates considered are either available in Supplementary Data 1 or can be calculated from genomewide variants (Geno_PC1, Geno_PC2, Geno_PC3). The percent of variance in expression explained by the PEER factors for expression and splicing phenotypes for all tissues. B.) Posterior variance of the PEER factor weights across the tissues for expression and splicing values.

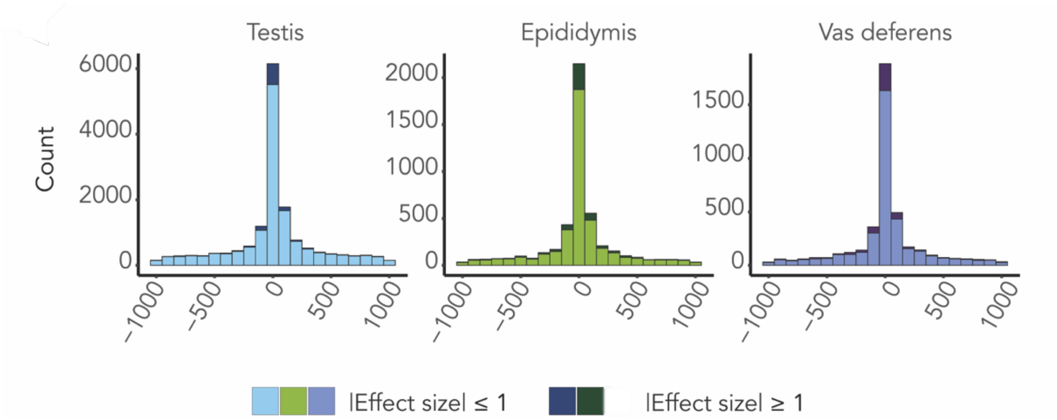

Supplementary Figure 6. eQTL and sQTL characteristics across the three tissues. Distribution of the distance of eQTL from the transcription start sites for all three reproductive tissues. Darker colors represent large effect eQTL (|slope_aFC >= 1|), while lighter colors represent small effect eQTL (|slope_aFC <= 1|).

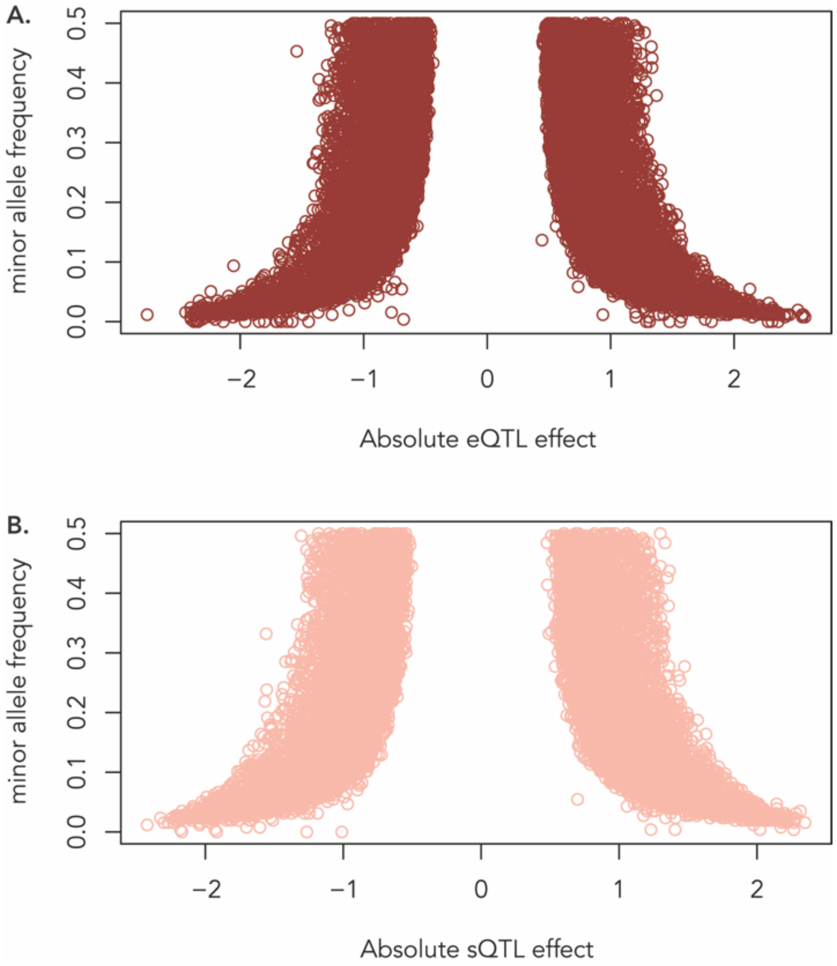

Supplementary Figure 7. Minor allele frequency and effect size of eQTL and sQTL. A.) Minor allele frequency and effect size of eQTL identified across the three tissues. B.) Minor allele frequency and effect size of sQTL identified across the three tissues.

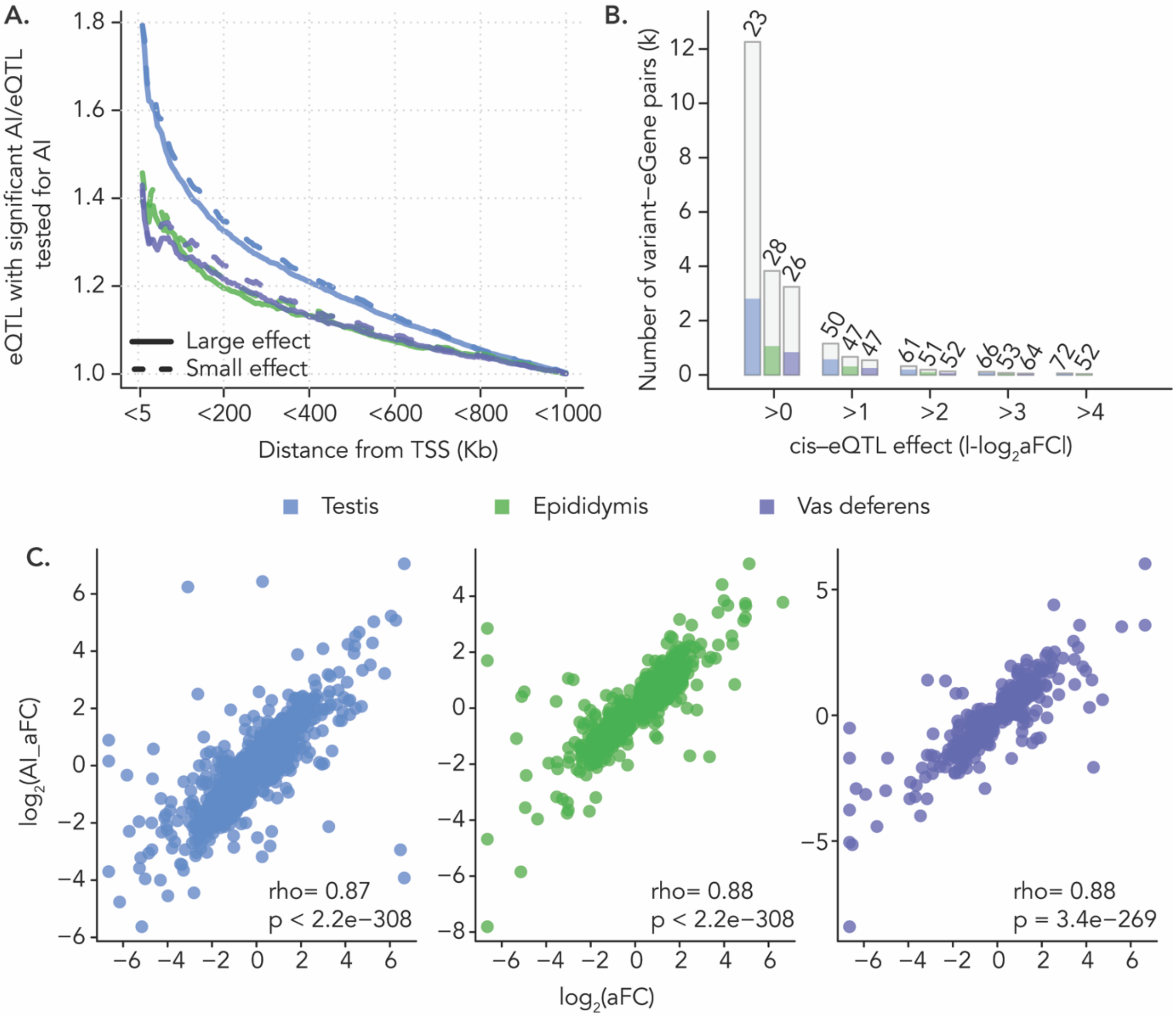

Supplementary Figure 8. Association between allelic imbalance (AI) and the expression of eGenes to assess cis-regulatory activity of linked eQTL. A.) Distribution of cis-eQTL associated with significant allelic imbalance grouped by their proximity to the transcription start site (TSS, in bins of 5 kb) in the three tissues. Data for large effect ($|\log_2$ slope_aFC$| >= 1$) and small effect ($|\log_2$ slope_aFC$| < 1$) eQTL are presented separately. B.) Number (in thousands) of variant-gene pairs tested for allelic imbalance (height of the bar) and number of variant-gene pairs with significant allelic imbalance (height of colored bar) grouped by their effect sizes ($|\log_2$ slope_FC$|$). The percentage of cis-eQTL with significant allelic imbalance are indicated at the top of the bar. C.) Correlation (Spearman's rho) between the cis-eQTL effect estimated as slope_aFC and effect estimated from the allelic imbalance data (AI_aFC). Only variant-gene pairs showing significant allelic imbalance (FDR <0.05) were considered. Spearman's correlations and their significances are indicated at the bottom.

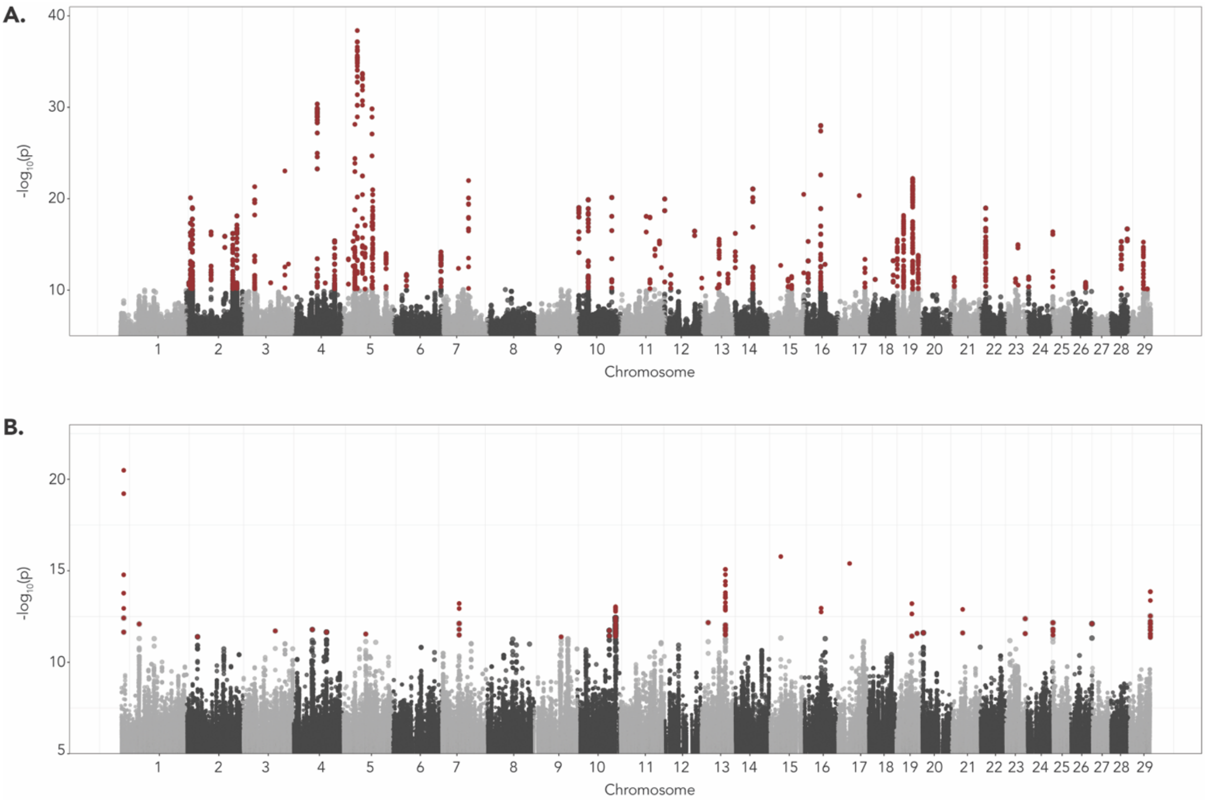

Supplementary Figure 9. Trans molQTL mapping in testis tissue. A) Manhattan plot representing the results of trans-eQTL mapping. B) Manhattan plot representing the results of trans-sQTL mapping. Red color indicates significant e/sVariants.

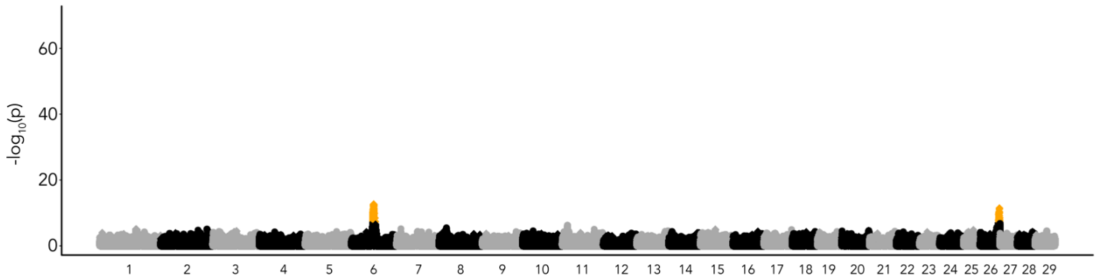

Supplementary Figure 10. Additive GWAS of male fertility. Manhattan plot showing the result from a mixed model-based genome-wide association between male fertility of 3736 bulls and 14,587,859 imputed sequence variants from additive association models. Orange dots indicate variants that are significantly associated with male fertility at a significance threshold of 5e-08.

A.

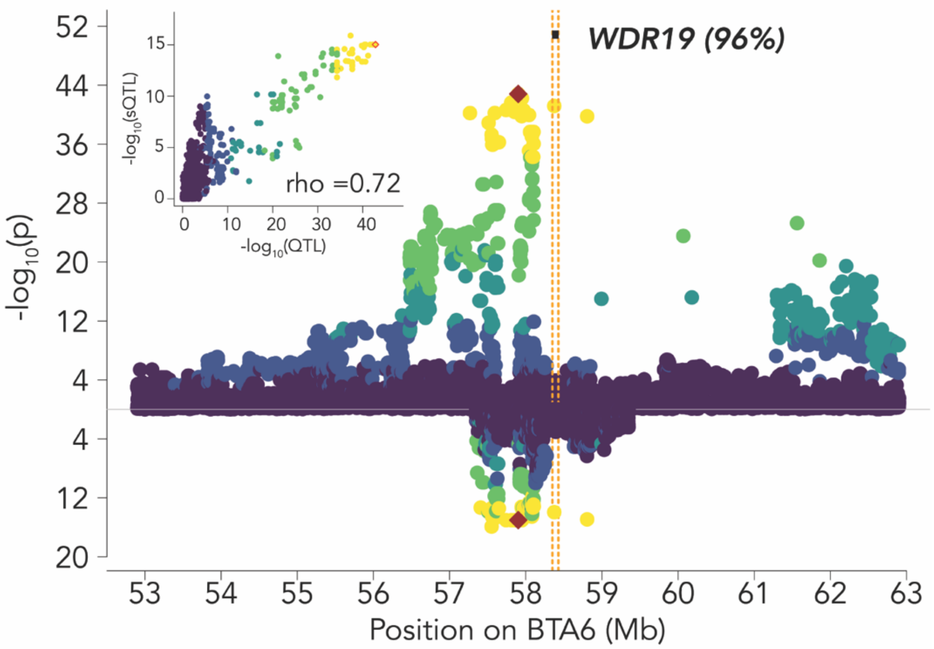

B.

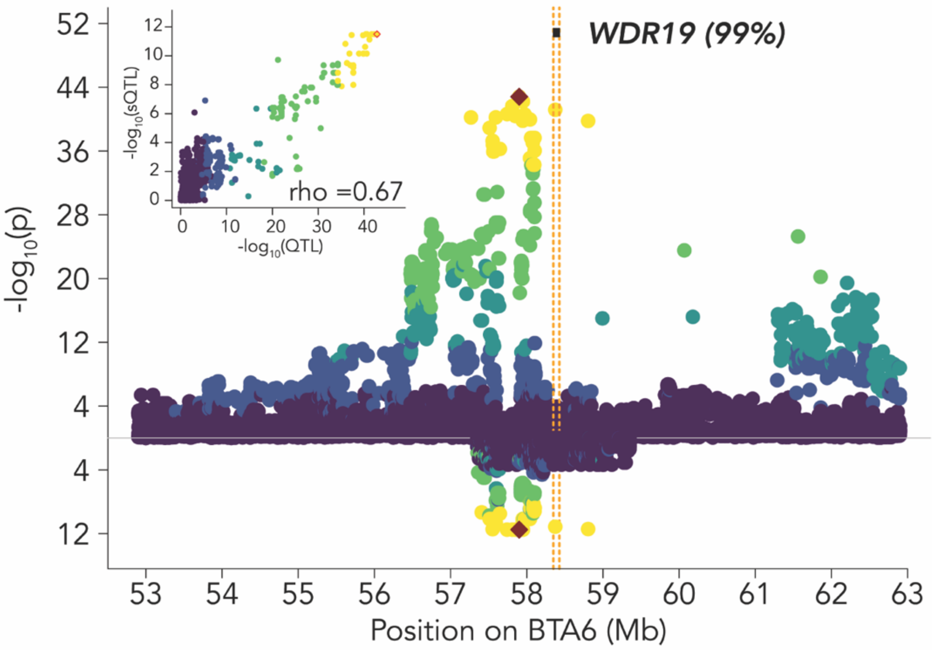

Supplementary Figure 11. Molecular *WDR19* phenotypes overlap with a male fertility QTL. A.) Mirrored plots of $-\log_{10}$(p)-values from association testing between imputed sequence variants on chromosome 6 and fertility in 3736 bulls (top) and splicing phenotypes for intron cluster 63814 spanning the splice junction 58,373,894-58,374,821 of the gene *WDR19* (bottom) in 84 epididymis samples (A) and intron cluster spanning the same splice junction of the gene WDR19 in 103 vas deferens samples (B). The inset plots show the correlation between GWAS and sQTL $-\log_{10}$(p)-values. Color indicates the linkage disequilibrium (R^2) between the most likely colocalized variant in the two tissues (6:57900948_G_A, red colored) and all other variants.

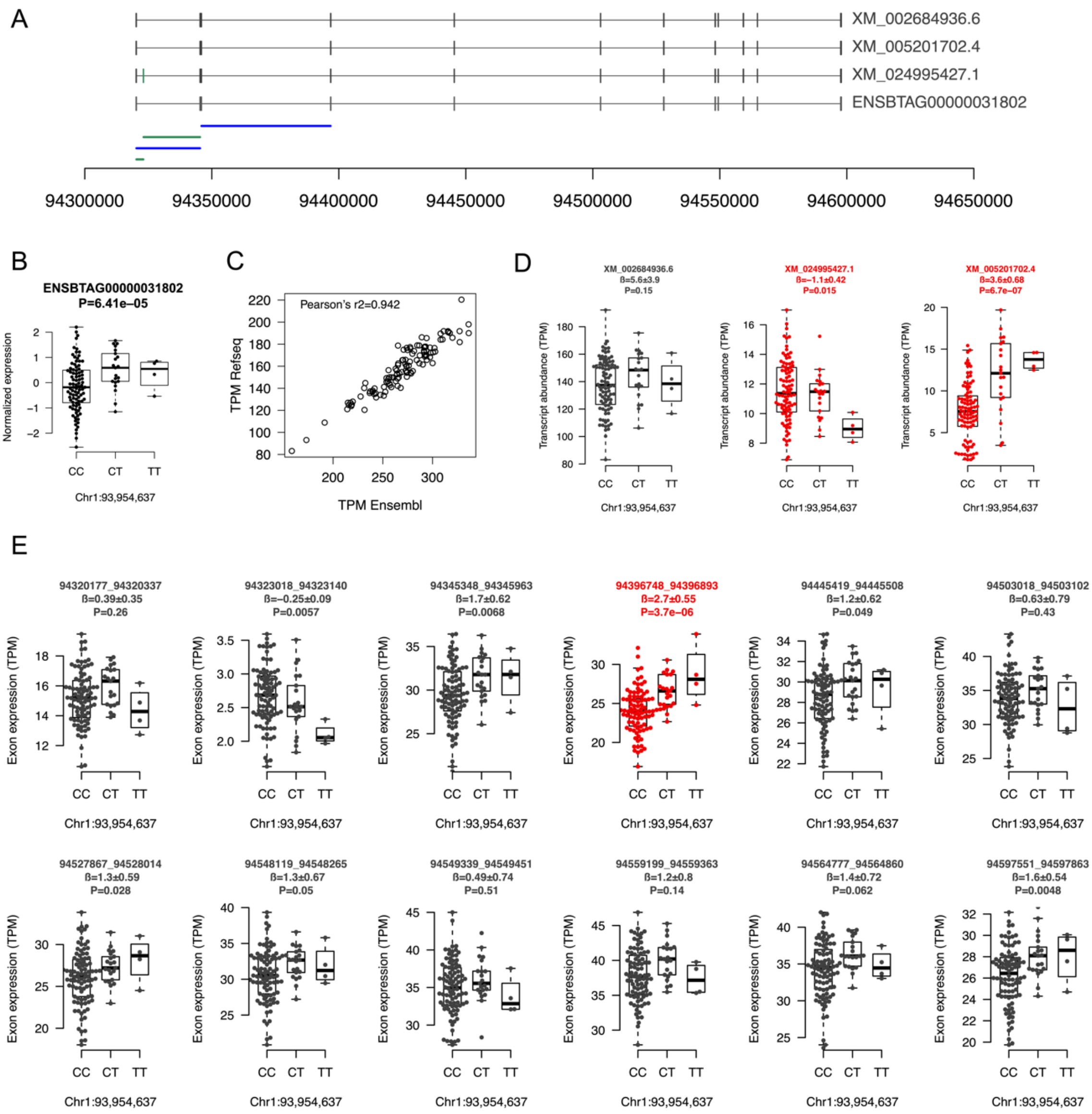

Supplementary Figure 12. Alternative splicing and expression of *SPATA16*. A) Comparison of the structure between three *SPATA16* isoforms (XM_002684936.6, XM_005201702.4, XM_024995427.1) that are annotated in Refseq (version 106) and the canonical *SPATA16* transcript (ENSBTAG00000031802) annotated from Ensembl (version 104). Boxes represent exons. The exon private to XM_024995427.1 is colored in green. Blue and green lines below the gene structures indicate the four splicing junctions of the significant intron cluster. Green color indicates two junctions that involve the exon private to XM_024995427.1. B) Effect of the Chr1:93954637 variant on the expression of *ENSBTAG00000031802*. The P value is from a linear regression of TPM values on the genotype (coded as 0, 1, 2) of 117 samples while accounting for age, RIN, 10 PEER factors and three principal components of a genomic relationship matrix. C) The gene-level TPM values of *SPATA16* are highly correlated (Pearson's rho=0.942) between the Ensembl and Refseq annotation. D) Effect of the Chr1:93954637 variant on transcript-level expression estimates. Transcripts are from the Refseq annotation. Red color indicates significant transcripts. E) Effect of the Chr1:93954637 variant on exon-level expression estimates. Exons are from the Refseq annotation. Red color indicates significant exons. The P values presented in D) and E) are from a linear regression of TPM values on the genotype (coded as 0, 1, 2) of 117 testis samples while accounting for age, RIN and three principal components of a genomic relationship matrix.

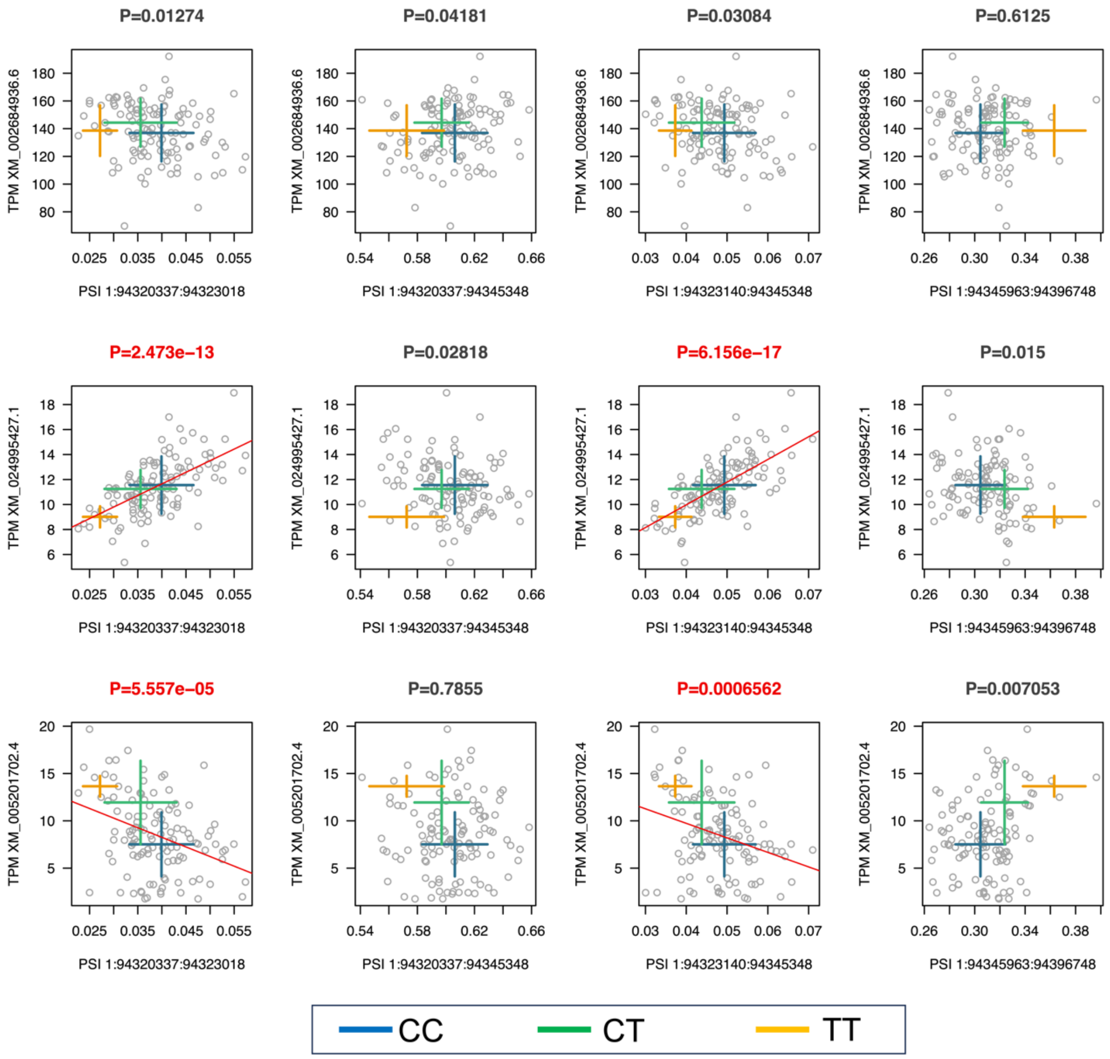

Supplementary Figure 13. *SPATA16* TPM and PSI correlations. Correlation between the expression (quantified in TPM) of the three *SPATA16* isoforms (XM_002684936.6, XM_005201702.4, XM_024995427.1) that are annotated in Refseq and the percent-spliced-in (PSI)-values of the four splice junctions of the significant intron cluster. P values were from a linear regression of TPM on PSI. Red color indicates significant transcript-splice junction relationships and the red line is the corresponding regression line. The two splice junctions that are significantly associated with the expression of XM_005201702.4 and XM_024995427.1 involve the exon private to XM_024995427.1. Different colors indicate mean ± standard deviations for PSI and TPM values for different Chr1:93954637 genotypes.

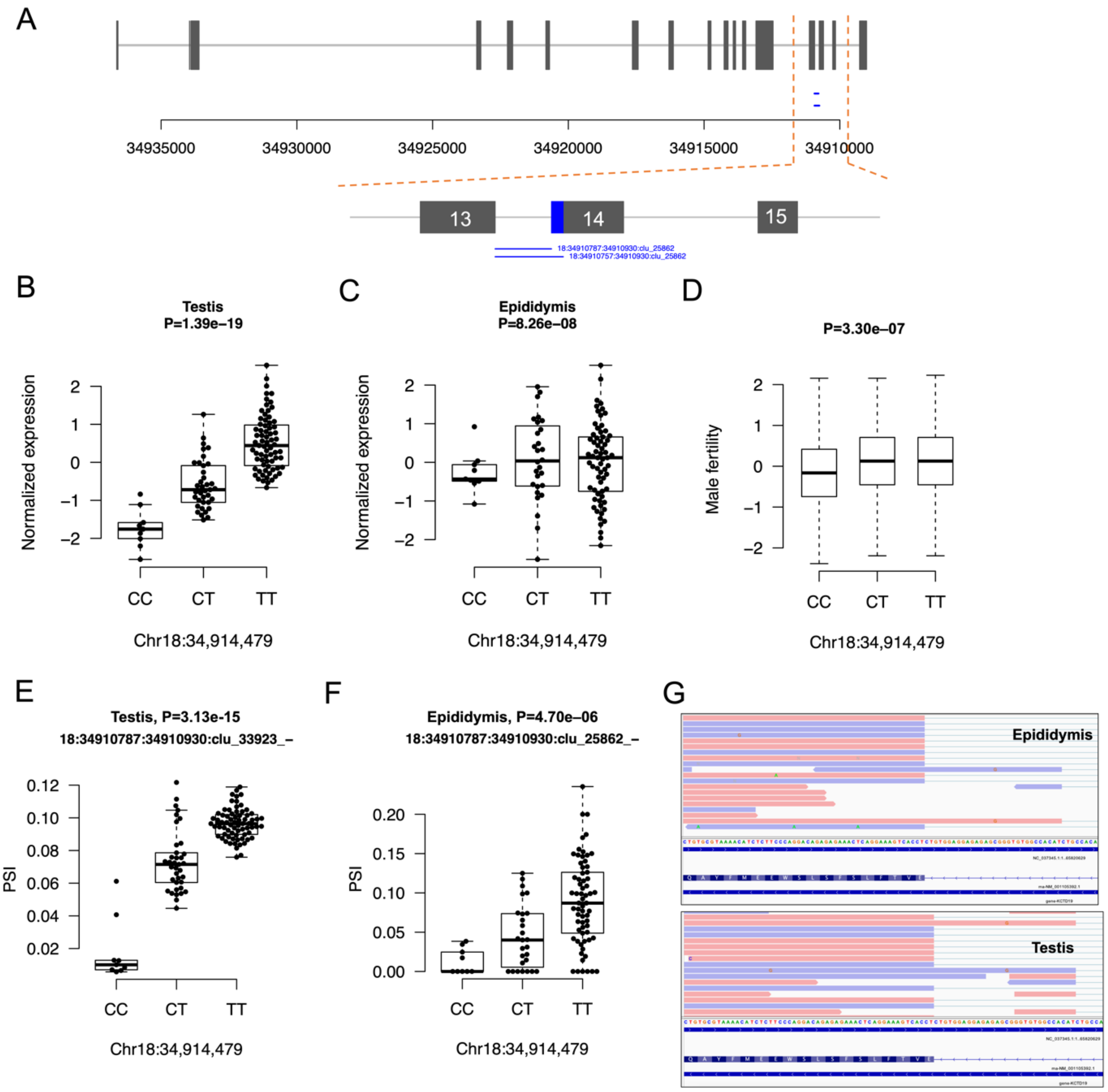

Supplementary Figure 14. Alternative splicing and expression of *KCTD19*. A) Structure of *KCTD19*. Boxes represent exons. Blue lines indicate two splice junctions within a significant intron cluster. The utilization of splice junction 34910787:34910930 adds 30 bases coding sequence to the 14th exon (indicated with the blue box preceding exon 14). B) and C) Effect of the Chr18:34914479 variant on the expression of *KCTD19* in testis and epididymis. P values are from the nominal eQTL mapping. D) Effect of the Chr18:34914479 variant on male fertility. The P value is from the non-additive GWAS model. E) and F) Effect of the Chr18:34914479 variant on *KCTD19* splicing in testis and epididymis. The P values are from the nominal sQTL mapping. G) Representative IGV screenshots of epididymis and testis RNA sequencing alignments validate the alternative start of exon 14.

Supplementary Table 1. Sequence variants considered after different filtering criteria were applied. Variants remaining after different filtering methods. Minor allele frequency (MAF) filters were applied separately for each subset of samples. HWE - Hardy-Weinberg equilibrium.

**# Variants after filtering**	**Number of samples (total)**	**No filtering**	**HWE and missingness**	**Imputation accuracy > 0.5**
	118	29,660,795	29,238,235	21,501,032
**MAF filters**	**Tissue**	**MAF ≥ 0.5%**	**MAF ≥ 1.0%**	**MAF ≥ 5.0%**
	Testis (117)	19,964,363	18,919,356	14,192,337
	Epididymis (103)	19,704,122	18,606,598	14,041,832
	Vas deferens (84)	21,041,956	19,367,827	14,208,661

Supplementary Table 2. RNA read alignment. Information on the amount of RNA sequence data available for the three tissues. The average and the standard deviation are reported for the RNA integrity number (RIN), the number of aligned reads, and the number of aligned reads after WASP filtering.

Tissue	RIN	# Aligned RNA reads	# Aligned RNA reads (WASP)
Testis	9.0 ± 0.5	257,050,199 ± 35,278,398	240,544,139 ± 32,870,352
Epididymis	8.4 ± 0.9	283,746,666 ± 35,375,776	267,645,035 ± 32,952,821
Vas deferens	5.9 ± 1.1	262,089,072 ± 25,546,308	251,031,216 ± 24,272,517

Supplementary Table 3. Types of expressed and spliced genes, and types of eGenes and sGenes. Breakdown of the number and proportion of expressed and spliced genes (after filtering), and eGenes and sGenes (after molQTL discovery), by type per tissue. "Other" includes pseudogenes and various small RNAs.

	Type	Testis	Epididymis	Vas deferens
**Expressed genes**	Protein coding	18,149 (89.7%)	17,815 (87.4%)	16,809 (88.2%)
	Long non-coding	933 (4.6%)	1,002 (4.9%)	831 (4.4%)
	Other	1,140 (5.7%)	1,559 (7.7%)	1,423 (7.4%)
**Spliced genes**	Protein coding	14,087 (95.7%)	13,470 (96.0%)	12,364 (96.7%)
	Long non-coding	603 (4.1%)	530 (3.8%)	389 (3.0%)
	Other	34 (0.2%)	26 (0.2%)	27 (0.3%)
**eGenes**	Protein coding	10,376 (93.0%)	3,974 (91.3%)	3,630 (93.3%)
	Long non-coding	497 (4.5%)	202 (4.7%)	159 (4.1%)
	Other	291 (2.5%)	171 (4.0%)	100 (2.6%)
**sGenes**	Protein coding	6,735 (96.2%)	2,571 (96.7%)	1,660 (96.7%)
	Long non-coding	247 (3.5%)	84 (3.1%)	51 (3.0%)
	Other	18 (0.3%)	7 (0.2%)	7 (0.3%)

Supplementary Table 4. Expression of differentially spliced genes. Median gene expression (TPM) for differentially spliced genes

	Testis	Epididymis	Vas deferens
11,474 genes with alternative splicing in all tissues	17.37	15.89	20.18
Genes with no alternative splicing	1.73 (5,523 genes)	1.91 (6,355 genes)	2.13 (6,271 genes)

References

1. Liu, S. *et al.* A multi-tissue atlas of regulatory variants in cattle. *Nat Genet* **54**, 1438–1447 (2022).
2. Robertson, M. J. *et al.* Large-scale discovery of male reproductive tract-specific genes through analysis of RNA-seq datasets. *BMC Biology* **18**, 103 (2020).
