# Supplementary material for: Molecular quantitative trait loci in reproductive tissues impact male fertility in cattle
Source: Nat Commun. 2024 Jan 22;15:674. doi: 10.1038/s41467-024-44935-7 (PMC10803364; doi:10.1038/s41467-024-44935-7)
Supplement: Supplementary file 3 — Description of Additional Supplementary Files [file 41467_2024_44935_MOESM3_ESM.pdf]

Description of Additional Supplementary Files:

Supplementary Data 1: Spread sheet containing metadata for the 118 samples. Accession numbers from the ENA for the DNA and RNA samples, as well as comprehensive metadata for the sample and each tissue.

Supplementary Data 2: Tissue specific and highly tissue enriched genes. Genes (top 100 ordered according to their expression) that are either tissue specific expressed (i.e., expressed in one tissue but not the other two) or show tissue-enriched expression (tissue-specificity index >50) in the three tissues.
