# Supplementary material for: Molecular quantitative trait loci in reproductive tissues impact male fertility in cattle
Source: Nat Commun. 2024 Jan 22;15:674. doi: 10.1038/s41467-024-44935-7 (PMC10803364; doi:10.1038/s41467-024-44935-7)
Supplement: Supplementary file 6 — Reporting Summary [file 41467_2024_44935_MOESM6_ESM.pdf]

nature portfolio

Corresponding author(s): Hubert Pausch

Last updated by author(s): 05 December 2023

Reporting Summary

Nature Portfolio wishes to improve the reproducibility of the work that we publish. This form provides structure for consistency and transparency in reporting. For further information on Nature Portfolio policies, see our Editorial Policies and the Editorial Policy Checklist.

Statistics

For all statistical analyses, confirm that the following items are present in the figure legend, table legend, main text, or Methods section.

n/a	Confirmed	
☐	☒	The exact sample size (*n*) for each experimental group/condition, given as a discrete number and unit of measurement
☐	☒	A statement on whether measurements were taken from distinct samples or whether the same sample was measured repeatedly
☐	☒	The statistical test(s) used AND whether they are one- or two-sided<br>*Only common tests should be described solely by name; describe more complex techniques in the Methods section.*
☐	☒	A description of all covariates tested
☐	☒	A description of any assumptions or corrections, such as tests of normality and adjustment for multiple comparisons
☐	☒	A full description of the statistical parameters including central tendency (e.g. means) or other basic estimates (e.g. regression coefficient) AND variation (e.g. standard deviation) or associated estimates of uncertainty (e.g. confidence intervals)
☐	☒	For null hypothesis testing, the test statistic (e.g. *F*, *t*, *r*) with confidence intervals, effect sizes, degrees of freedom and *P* value noted<br>*Give P values as exact values whenever suitable.*
☒	☐	For Bayesian analysis, information on the choice of priors and Markov chain Monte Carlo settings
☒	☐	For hierarchical and complex designs, identification of the appropriate level for tests and full reporting of outcomes
☐	☒	Estimates of effect sizes (e.g. Cohen's *d*, Pearson's *r*), indicating how they were calculated

Our web collection on statistics for biologists contains articles on many of the points above.

Software and code

Policy information about availability of computer code

Data collection	Standard code from sequencing providers (Illumina) was used to gather sequencing data.
Data analysis	Version numbers and references for all software and scripts used to process and analyse data are given also in the manuscript. The following software were used:<br>PANTHER (v17.0, http://geneontology.org/), Ensembl BioMART (http://www.ensembl.org/biomart/martview), fastp (v0.19.4), Sambamba (v0.6.6), BWA (v0.717), Picard tools (v2.25.7), mosdepth (v0.3.6), DeepVariant (v1.3), GLnexus (v1.4.1), Beagle (v4.1), Plink (v1.9), QTLtools (v1.3.1), STAR (version 2.7.9a), featureCounts (v2.0.3), dendextend (v1.16.0), Regtools (v0.5.2), LeafCutter (v0.2.9), DESeq2 (v3.18), VEP (v109.3), Beagle (v5.4), phASER (v1.2.0), RepeatMasker (v4.1.4), GenMap (v1.3.0), crossmap (v1.2), Mashr (v0.2.69), GCTA (v1.92.1), S-PrediXcan (v0.7.5), coloc (5.1.0.1), kallisto (v0.46.1), cluster_prepare_fastqtl.py (https://github.com/broadinstitute/gtex-pipeline/tree/master/qtl/leafcutter/src/cluster_prepare_fastqtl.py), map_clusters_to_genes.R (https://github.com/broadinstitute/gtex-pipeline/tree/master/qtl/leafcutter/src/map_clusters_to_genes.R), collapse_annotation.py (https://github.com/broadinstitute/gtex-pipeline/tree/master/gene_model/collapse_annotation.py), gtex_tiss_chrom_training.R (https://github.com/hakyimlab/PredictDB_Pipeline_GTEx_v7/blob/master/model_training/scripts/gtex_tiss_chrom_training.R), gdc-fastq-splitter (https://github.com/kmhernan/gdc-fastq-splitter)

For manuscripts utilizing custom algorithms or software that are central to the research but not yet described in published literature, software must be made available to editors and reviewers. We strongly encourage code deposition in a community repository (e.g. GitHub). See the Nature Portfolio guidelines for submitting code & software for further information.

Data

Policy information about availability of data

All manuscripts must include a data availability statement. This statement should provide the following information, where applicable:

- Accession codes, unique identifiers, or web links for publicly available datasets
- A description of any restrictions on data availability
- For clinical datasets or third party data, please ensure that the statement adheres to our policy

DNA and RNA sequencing data of 118 bulls are available in the ENA database at the study accessions PRJEB28191 (https://www.ebi.ac.uk/ena/browser/view/PRJEB28191) and PRJEB46995 (https://www.ebi.ac.uk/ena/browser/view/PRJEB46995). Comprehensive metadata for all samples are available as Supplementary Data 1. Gene expression and splicing matrices, a VCF file of genome-wide genotypes used for e/sQTL mapping, a cross-table to link genotype and transcriptome data, as well as summary statistics used for transcriptome-wide association testing have been archived at zenodo (https://zenodo.org/records/10053815). Transcriptomic data from the cattle GTEx consortium are available at zenodo (https://zenodo.org/records/7560235). Human and mice reproductive tract specific genes are available in Table S5 and Table S6 from Robertson et al.31. Human testis specific genes are available in Supporting Table 4 from Djureinovic et al.29. The bovine reference sequence ARS-UCD1.2 is available at https://www.ncbi.nlm.nih.gov/datasets/genome/GCF_002263795.1/. The Ensembl gene annotation (release 104) is available at https://ftp.ensembl.org/pub/release-104/gtf/bos_taurus/. The Refseq gene annotation (version 106) is available at ftp://ftp.ncbi.nlm.nih.gov/refseq/B_taurus/annotation_releases/106/GCF_002263795.1_ARS-UCD1.2/GCF_002263795.1_ARS-UCD1.2_genomic.gff.gz. Raw data to reproduce the figures are provided in the Source Data file.

Research involving human participants, their data, or biological material

Policy information about studies with human participants or human data. See also policy information about sex, gender (identity/presentation), and sexual orientation and race, ethnicity and racism.

Reporting on sex and gender	na
Reporting on race, ethnicity, or other socially relevant groupings	na
Population characteristics	na
Recruitment	na
Ethics oversight	na

Note that full information on the approval of the study protocol must also be provided in the manuscript.

Field-specific reporting

Please select the one below that is the best fit for your research. If you are not sure, read the appropriate sections before making your selection.

[x] Life sciences [ ] Behavioural & social sciences [ ] Ecological, evolutionary & environmental sciences

For a reference copy of the document with all sections, see nature.com/documents/nr-reporting-summary-flat.pdf

Life sciences study design

All studies must disclose on these points even when the disclosure is negative.

Sample size	No statistical method was applied to predetermine sample size. Our sample is in the rage of typical eQTL studies (e.g.,https://academic.oup.com/nar/article/46/22/e133/5090771). Cattle breeds such as Braunvieh have a low effective population size (Ne ~70), so less individuals are required to capture genetic diversity than in e.g., human populations.
Data exclusions	The fastp software was used to exclude low quality reads and bases; no other data were excluded
Replication	This was an exploratory study with an aim to investigate the transcriptional complexity of three reproductive tissues. No replication was applied. Replication would involve collecting a similar number of transcriptomes, which is costly prohibitive.
Randomization	Tissue was sampled from randomly selected animals. This study did not involve experimental groups. PEER factors were estimated to account for possible batch effects.
Blinding	This study did not involve allocating samples to different groups.

Reporting for specific materials, systems and methods

We require information from authors about some types of materials, experimental systems and methods used in many studies. Here, indicate whether each material, system or method listed is relevant to your study. If you are not sure if a list item applies to your research, read the appropriate section before selecting a response.

Materials & experimental systems

n/a	Involved in the study
☒	☐ Antibodies
☒	☐ Eukaryotic cell lines
☒	☐ Palaeontology and archaeology
☐	☒ Animals and other organisms
☒	☐ Clinical data
☒	☐ Dual use research of concern
☒	☐ Plants

Methods

n/a	Involved in the study
☒	☐ ChIP-seq
☒	☐ Flow cytometry
☒	☐ MRI-based neuroimaging

Animals and other research organisms

Policy information about studies involving animals; ARRIVE guidelines recommended for reporting animal research, and Sex and Gender in Research

Laboratory animals	Bulls from the Braunvieh breed were included
Wild animals	This study did not involve wild animals
Reporting on sex	Only male animals were considered as «male reproduction» was the focus of the paper
Field-collected samples	Tissue was collected at a commercial abattoir.
Ethics oversight	Ethical oversight was not needed for this study, as the tissue was collected from random animals at a commercial abattoir. The following ethics statement is included at the beginning of the Methods section: «Tissue of male Bos taurus taurus animals was sampled at a commercial abattoir. The decision to slaughter the bulls was independent from our study. None of the authors of the present study were involved in the decision to slaughter the bulls. No ethics approval was required for this study. ».

Note that full information on the approval of the study protocol must also be provided in the manuscript.

Plants

Seed stocks	na
Novel plant genotypes	na
Authentication	na
